# Effect of rabbit doe-litter separation on 24-hour changes of luteinizing hormone, follicle stimulating hormone and prolactin release in female and male suckling pups

**DOI:** 10.1186/1477-7827-3-50

**Published:** 2005-09-27

**Authors:** Pilar Cano, Vanesa Jiménez-Ortega, Maria P Álvarez, Mario Alvariño, Daniel P Cardinali, Ana I Esquifino

**Affiliations:** 1 Departamento de Bioquímica y Biología Molecular III, Facultad de Medicina, Universidad Complutense de Madrid, Spain; 2 Departamento de Biología Celular, Facultad de Medicina, Universidad Complutense de Madrid, Spain; 3 Departamento de Producción Animal, E.T.S.I. Agrónomos, Universidad Politécnica de Madrid, Spain; 4 Departamento de Fisiología, Facultad de Medicina, UBA, Buenos Aires, Argentina

## Abstract

**Background:**

The daily pattern of nursing of the rabbit pup by the doe is the most important event in the day for the newborn and is neatly anticipated by them. Such anticipation presumably needs a close correlation with changes in hormones that will allow the pups to develop an appropriate behavior. Although a number of circadian functions have been examined in newborn rabbits, there is no information on 24-h pattern of gonadotropin release or on possible sex-related differences in gonadotropin or prolactin (PRL) release of pups. This study examined the 24-h changes of plasma luteinizing hormone (LH), follicle stimulating hormone (FSH) and prolactin (PRL) in 11 days old suckling female and male rabbits left with the mother or after short-term (i.e., 48 h) doe-litter separation.

**Methods:**

Animals were kept under controlled light-dark cycles (16 h – 8 h; lights on at 08:00 h). On day 9 post partum, groups of 6–7 female or male rabbit pups were separated from their mothers starting at 6 different time intervals in the 24 h cycle. Pups were killed 48 h after separation. At each time interval groups of male or female pups that stayed with the mother were killed as controls. Plasma, LH, FSH and PRL levels were measured by specific radioimmunoassays.

**Results:**

In pups kept with their mother plasma FSH and LH maxima occurred at the first and second part of the light phase (at 13:00 and 17:00 – 21:00 h, respectively) (females) or as two peaks for each of the hormones (at 13:00 and 01:00 h) (males). PRL release was similar in female and male rabbit pups kept with their mother, showing a 24-h pattern with two peaks, at 13:00 and 01:00 h, respectively. Mean 24-h values of gonadotropins and PRL did not differ between sexes. Isolation of pups for 48 h augmented circulating gonadotropin and PRL levels and distorted hormone 24-h pattern to a similar extent in both sexes.

**Conclusion:**

Significant sex differences in 24-h changes in LH and FSH, but not in PRL, release occurred in rabbit pups kept with the doe. Separation of newborn pups from their mother augmented circulating gonadotropin and PRL levels and disrupted 24-h rhythmicity of gonadotropin and PRL release similarly in both sexes. The effect of pups' isolation can be attributed either to a modification of the circadian pacemaker or to a masking effect on some of its output overt rhythms.

## Background

In contrast with an elaborate nest-building process, following parturition, maternal care in rabbits is restricted to a single, brief (around 3–5 min) nursing bout per day, an activity that is displayed with circadian periodicity during the dark phase of daily photoperiod (around 02:00 h) [1]. Despite the short duration of each nursing bout, the altricial rabbit pups (which are blind for the first 10 days of life) can locate the mother's nipples and suckle milk due to the perception of an olfactory signal that is emitted from the mother's ventrum [2].

Thus the daily pattern of nursing of the rabbit pup by the doe is the most important event in the day of newborn and is neatly anticipated by them [3,4]. The newborn rabbits spend most of the time between feeds lying quietly together, becoming increasingly active and gradually exposed 1–2 h in advance to nursing. These events presumably need a close correlation with changes in hormones that will allow the pups to develop an appropriate behavior. In fact, an earlier development of the circadian secretory pattern of prolactin (PRL) occurs in infantile rabbits [5,6] as compared to other laboratory species e.g., the albino rat [7,8]. Although a number of circadian functions have been examined in newborn rabbits [1,9-12] there is no information on 24-h pattern of gonadotropin release nor on possible sex-related differences in gonadotropin or PRL release of pups.

When litters are separated from their mother and deprived of one nursing they display the usual pattern of anticipatory behavior on the first day of separation, but when the doe fails to arrive, gradually become less active and covered over again with nest material [1]. The following day, approximately 45–48 h after last nursing, the pups again become aroused, uncovered and are able to suckle normally. Therefore, after 48-h doe-litter separation, rabbit pups exhibit a usual daily pattern of behavior, as compared to controls.

The present study was designed to answer the following questions: (1) are there sex differences in gonadotropin and PRL release in rabbit pups?; (2) does separation from the mother modify gonadotropin and PRL release in pups? To achieve this, the 24-h changes in plasma LH, FSH and PRL were examined in 11 days old suckling female and male rabbits left with the mother or after short-term (i.e., 48 h) doe-litter separation.

## Results

**Figure 1:**
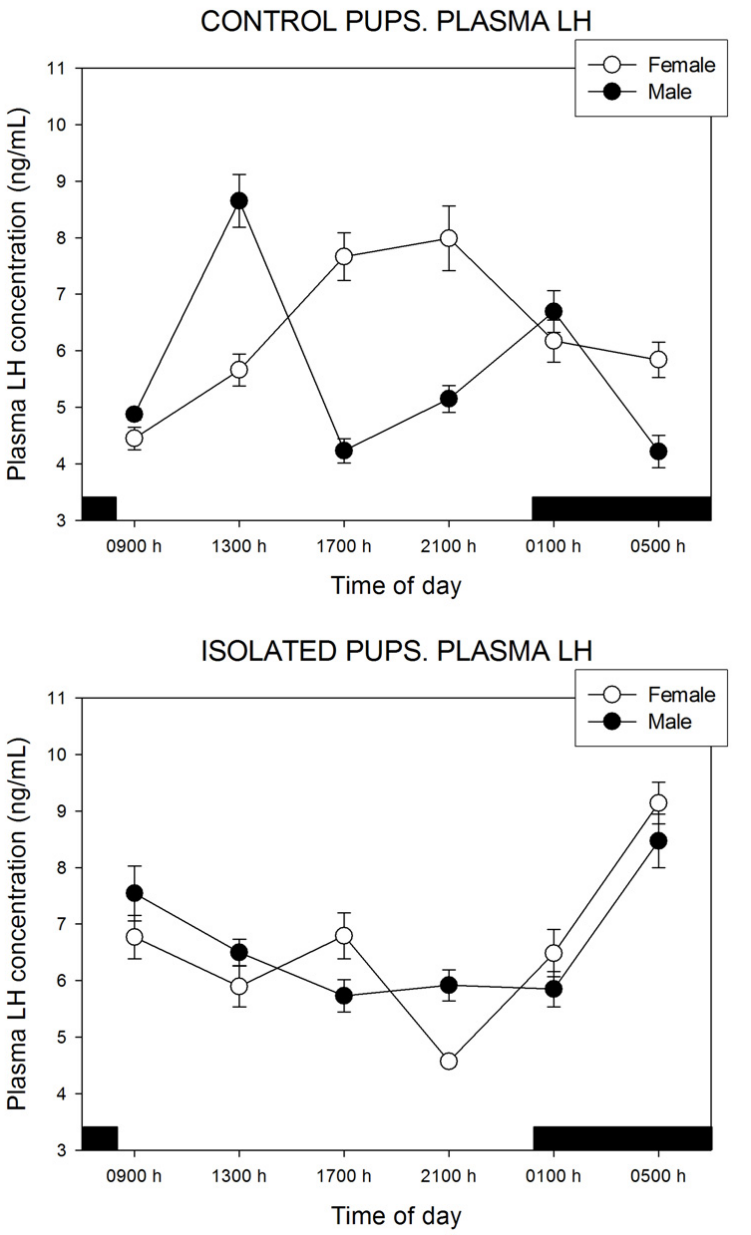
24-Hour changes in plasma LH levels in 11 days old rabbit pups kept with their mother or isolated for 48 h. Groups of 6–7 female or male pups were killed by decapitation at 6 different time intervals throughout a 24 h cycle. Bar indicates scotophase duration. Results are the means ± SEM. For statistical analysis, see text.

Figure 1 shows the plasma levels of LH throughout the day in female and male pups kept with or separated from the doe. A multivariate ANOVA of the whole set of observations indicated significant effects of treatment (F = 13.8, p < 0.0001) and of time of day (F = 4.1, p < 0.02), when assessed as main factors. Mean values of LH were 6.13 ± 1.37 and 6.97 ± 1.57 ng/mL (SD) in control and isolated females (p < 0.02, Student's t test) and 5.64 ± 1.71 and 6.53 ± 1.22 ng/mL in control and isolated males (p < 0.03, Student's t test). Significant interactions of "gender × time of day" (F = 10.9, p < 0.0001), "treatment × time of day" (F = 28.1, p < 0.0001), "treatment × gender" (F = 7.5, p < 0.007) and "treatment × time of day × gender" (F = 6.6, p < 0.001) were found. In female pups kept with their mother, plasma LH peaked at the second part of the light phase of daily photoperiod whereas two peaks were observed in males, i.e., at 13:00 and 01:00 h, respectively. Pup isolation from the mother distorted the circulating LH rhythm in a similar way in both sexes, exhibiting a peak at the second part of the scotophase (at 05:00 h) (Fig. 1).

**Figure 2:**
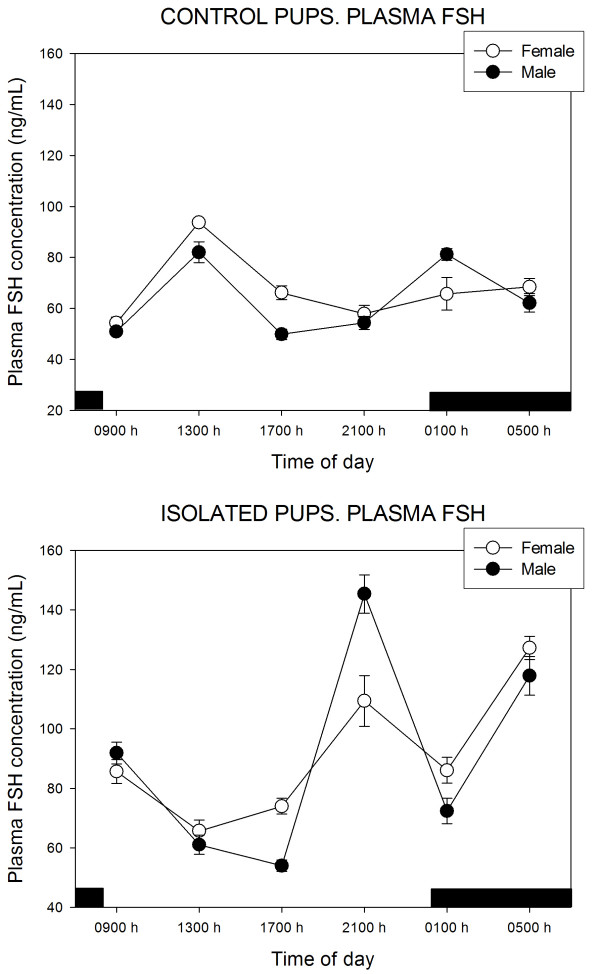
24-Hour changes in plasma FSH levels in 11 days old rabbit pups kept with their mother or isolated for 48 h. Groups of 6–7 female or male pups were killed by decapitation at 6 different time intervals throughout a 24 h cycle. Bar indicates scotophase duration. Results are the means ± SEM. For statistical analysis, see text.

Figure 2 depicts the circulating FSH concentration. A multivariate ANOVA showed significant effects of treatment (F = 155.3, p < 0.0001) and time of day (F = 24.2, p < 0.0001). Mean values of FSH were 66.1 ± 13.6 and 89.9 ± 22.6 ng/mL in control and isolated females (p < 0.001, Student's t test) and 63.6 ± 15.2 and 95.5 ± 38.2 ng/mL in control and isolated males (p < 0.001, Student's t test). Significant interactions of "gender × time of day" (F = 4.9, p < 0.0001), "treatment × time of day" (F = 48.4, p < 0.0001), and "treatment × time of day × gender" (F = 4.3, p < 0.01) were detected. In female pups kept with their mother, plasma FSH peaked at 13:00 h while two peaks were seen in males, i.e., at 13:00 and 01:00 h, respectively. Doe-litter separation distorted pups' plasma FSH rhythm to a similar extent in both sexes, with peaks at 09:00, 21:00 and 05:00 h. At certain time points, i.e. at 17:00 and 21:00 h, significant sex differences were seen (Fig. 2).

**Figure 3:**
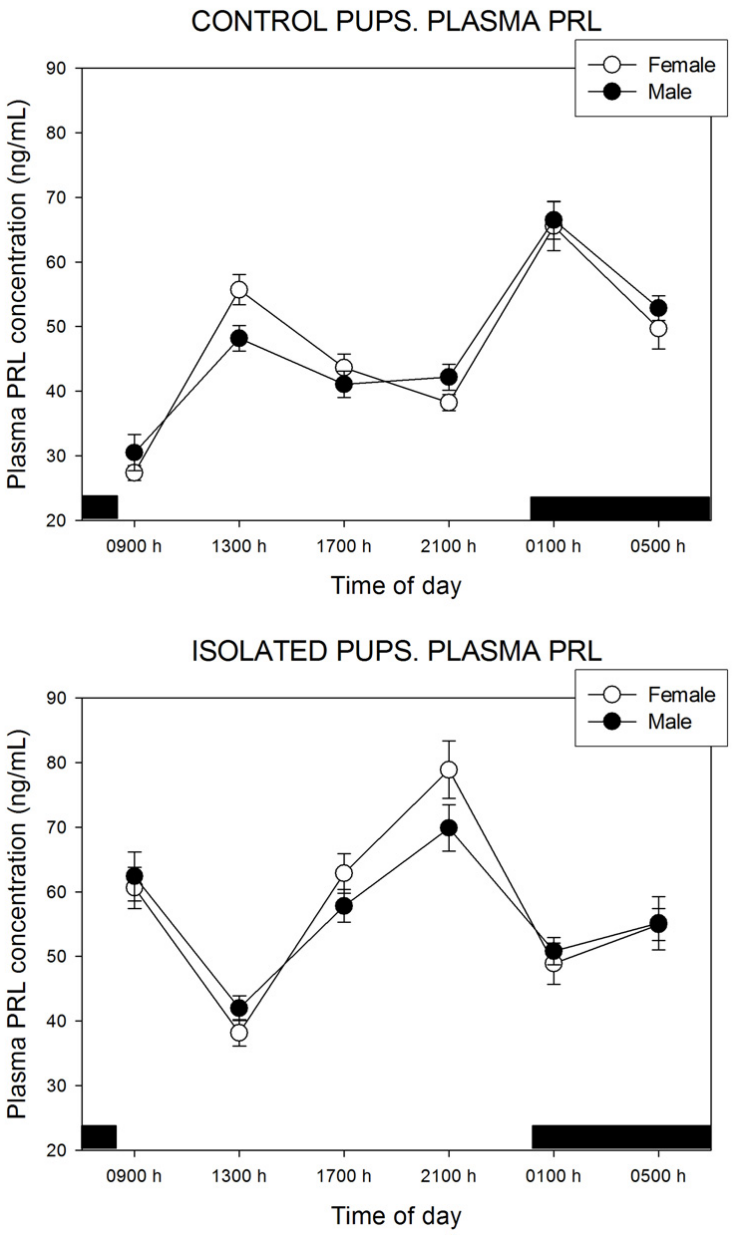
24-Hour changes in plasma PRL levels in 11 days old rabbit pups kept with their mother or isolated for 48 h. Groups of 6–7 female or male pups were killed by decapitation at 6 different time intervals throughout a 24 h cycle. Bar indicates scotophase duration. Results are the means ± SEM. For statistical analysis, see text.

Plasma PRL levels in control pups, and their responses to the separation from the doe, are depicted in Fig. 3. In the multivariate ANOVA the effects of treatment (F = 51.1, p < 0.0001) and of time of day (F = 9.3, p < 0.0001) were significant. Mean values of PRL were 46.6 ± 14.1 and 55.6 ± 12.2 ng/mL in control and isolated females (p < 0.01, Student's t test) and 47.6 ± 11.1 and 56.8 ± 12.4 ng/mL in control and isolated males (p < 0.002, Student's t test). PRL release was essentially similar in control female and male rabbit pups, i.e., a 24-h pattern with two peaks, at 13:00 and 01:00 h, respectively. The only significant interaction detected in the multivariate ANOVA was that of "treatment × time of day" (F = 37.9, p < 0.0001), namely, the isolation of pups brought about a significant increase of plasma PRL as well as a phase-advance of 3 h in both peaks (Fig. 3).

## Discussion

The questions posed in the Introduction can now be answered: (1) significant sex differences in 24-h rhythmicity of LH and FSH, but not of PRL release, occurred in rabbit pups kept with the doe; (2) separation of newborn pups from their mother augmented circulating gonadotropin and PRL levels and disrupted 24-h rhythmicity of their release to a similar way in both sexes.

Foregoing results document the existence of sex differences in 24-h rhythmicity of gonadotropin but not of PRL release in newborn rabbits. Mean value of gonadotropin and PRL release did not differ between sexes in rabbit pups. This is in contrast with data obtained in newborn rats demonstrating that newborn female rats have significantly higher plasma FSH and LH levels than their male counterparts [13]. Indeed, the crucial difference between a reflex ovulator (like the rabbit) and a spontaneous ovulator (like the rat) is that the former lacks the capacity to generate the gonadotropin ovulatory surge in response to estrogen or progesterone injection [14,15]. It is supposed that reflex ovulators have the genetic information for the organization of a cyclic gonadotropin releasing mechanism but its development is hindered by the action of sex steroids produced by the fetal gonads [16]. If this tenet is true, the differences between male and female rabbit pups would be less defined than in rodents, as found in the present study. That the process of defeminization of the female brain by perinatal gonadal steroids occurs in rabbits is indicated by studies in pregnant female rabbits treated with testosterone propionate from day 17–29 of gestation [17]. While there was no apparent effect on gestation and maternal behaviors of injected mothers, the female offspring showed anatomical and behavioral masculinization.

Healthy rabbit pups can, and often do, miss a feed and survive. Thus, separation of litters from their mother provides an appropriate model for investigating the ontogeny of gonadotropin and PRL 24-h rhythms in view that maternal influence can be nullified for a short period without disrupting the normal neonatal environment [1,18,19]. The foregoing results indicate that separation of litters from their mother augmented circulating mean gonadotropin and PRL levels. It also distorted circulating gonadotropin and PRL 24-h rhythms to a similar extent in both sexes.

In previous studies we examined some of the neuroendocrine mechanisms associated with the 24-h plasma PRL rhythm in male and female rabbit pups [5,6]. The PRL rhythm reported in control male and female pups herein is similar to that reported previously.

Circadian rhythms of developing mammals can be entrained by the rhythmicity of their mother [20,21]. Previous data demonstrated that when litters remained separated from the doe and deprived of two nursings they show the usual pattern of anticipatory behavior and at the appropriate time [1,18,19]. This occurs regardless of the significant changes in gonadotropin and PRL release demonstrated herein. Indeed, after doe-litter separation for 48 h, the rabbit pups show a normal daily pattern of behavior but a disrupted 24-h variation of plasma LH, FSH and PRL release and a significant increase in their plasma concentration, indicating a certain degree of internal desynchronization produced by the isolation procedure. It must be noted that the experimental design employed does not allow discerning whether the circadian phase of separation initiation is critical or doe-litter separation affects circadian profile of hormone release regardless time of day of separation. Studies including a larger cohort of pups all separated at one circadian time point, with subgroups sacrificed at different time intervals later will be helpful to clarify this point.

Temporal organization is an important feature of the biological systems and its main function is to facilitate adaptation of the organism to the environment [22,23]. Stress is capable of perturbing this temporal organization by affecting the shape and amplitude of a rhythm or by modifying the intrinsic oscillatory mechanism itself. In particular, social stress in rodents has been found to cause disruptions of the circadian rhythms of body temperature, heart rate, locomotor activity and hormone release [24-28]. The present results confirm previous studies in rodents showing that maternal separation evokes marked alteration in neuroendocrine control mechanisms also in rabbits [29]. Further experiments are needed to assess whether the changes in mean value as well in timing of 24-h rhythms of gonadotropins and PRL seen in isolated rabbit pups can attributed the effect of stress on either the endogenous clock that modulates the circadian variation of hormone release or via an interfering ("masking") effect on some output(s) of the clock. Additionally, to what extent the increase of stress hormones like ACTH and glucorticoids vary in a circadian pattern after isolation should be examined to provide a basis for physiological explanation of the phenomenological data hereby presented.

## Methods

### Animals

This study was performed using 84 multiparous, lactating Californian × New Zealand White crossbreed female rabbits. Animals were housed in the research facilities of the Departamento de Producción Animal, Universidad Politécnica de Madrid. They were maintained under controlled light-dark cycles (16 h – 8 h; lights on at 08:00 h), housed in individual metal cages, fed at libitum using a commercial pellet diet [Lab Rabbit Chow, Purina Mills, Torrejón de Ardoz, Madrid, Spain] and had free access to tap water. On day 1 after parturition, litter size was standardized to 8–9 by adding or removing kits to assure similar lactation conditions during the experiment. Nursing visit of does under these conditions occurred during the dark phase of the photoperiod (around 02:00 am) for 3–5 min [1], this fact being observed under our experimental conditions. The does and the pups that did not observed this feeding schedule were not used for this study. On day 9 post partum, groups of 6–7 female or male rabbit pups were separated from their mothers starting at different time intervals in the 24 h cycle, i. e., at 09:00, 13:00, 17:00, 21.00, 01:00 or 05:00 h. Pups were killed 48 h after separation from their mothers. At each time interval groups of male or female pups that stayed with the mother were killed as controls. The study was performed according to the CEE Council Directive [86/609, 1986] for the care of experimental animals.

### Hormone assay

Plasma, LH, FSH and PRL levels were measured by specific RIA methods [30] using AFP-3120489, AFP-472176 and AFP-991086 antibodies for, LH, FSH and PRL, respectively, as supplied by the National Institute of Health [NIH, Bethesda, MD, USA] and Dr. A.F. Parlow [Harbour-UCLA Medical Center, CA, USA]. The antibody titers used were, 1:250000 for LH, 1:45000 for FSH and 1:62500 for PRL assays, respectively. The volume of plasma used was 100 μL for LH, 75 μL for FSH and 10 μL for PRL. *Staphylococcus aureus *was used to precipitate the bound fraction. The assays were previously validated in our laboratory [30]

All samples were measured in the same assay run to avoid inter-assay variations. The limit of detection for LH, FSH and PRL was 0.05, 0.48 and 0.125 ng/mL respectively. The intra-assay coefficient of variation, calculated using a pool of plasma measured ten times in the same assay, was <5%.

### Statistics

After determining that the homogeneity-of-variance assumption was tenable and that the distribution appeared unimodal and nonskewed, the statistical analysis of results was performed by a multifactorial factorial analysis of variance (ANOVA). Generally, the multifactorial ANOVA included assessment of the treatment effect (i.e. the occurrence of differences in mean values between control and separated groups), of sex effects (differences in mean values between female and male pups), of time of day effects (the occurrence of daily changes) and of the interactions among the three factors (from which inference about differences in timing and amplitude could be obtained). Student's t tests to compare 24 h mean serum hormone values were performed when appropriate. P values lower than 0.05 were considered evidence for statistical significance.
